# Interleukin-8/CXCL8 is a growth factor for human lung cancer cells

**DOI:** 10.1038/sj.bjc.6602227

**Published:** 2004-11-16

**Authors:** Y M Zhu, S J Webster, D Flower, P J Woll

**Affiliations:** 1 Department of Clinical Oncology, Institute for Cancer Studies, University of Sheffield Medical School, Beech Hill Road, Sheffield S10 2RX, UK

**Keywords:** interleukin-8/CXCL8, CXCR1, CXCR2, lung cancer

## Abstract

Interleukin-8/CXCL8 (IL-8) is a chemokine and angiogenic factor. Recently, IL-8 was identified as an autocrine growth factor in several human cancers. Here, we investigated the expression and function of IL-8 in lung cancer cells. The expressions of IL-8 and its receptors, CXCR1 and CXCR2, were examined in a panel of non-small cell lung cancer (NSCLC) and small cell lung cancer (SCLC) cell lines. Using reverse transcription–polymerase chain reaction (RT–PCR) and enzyme-linked immunosorbent assay, we found that all NSCLC cell lines tested produced modest or high levels of IL-8 (up to 51 ng ml^−1^ 10^6^ cells^−1^). Expression of CXCR1 and CXCR2 was found by RT–PCR and flow cytometry in two out of three cell lines. In contrast, SCLC cell lines produced very low or undetectable levels of IL-8, but expressed CXCR1 and CXCR2. We next investigated whether IL-8 could act as an autocrine growth factor in two NSCLC cell lines (H460 and MOR/P) expressing both IL-8 and its receptors. We found that cell proliferation was attenuated by anti-IL-8 neutralising antibody to 71 and 76% in H460 and MOR/P, respectively (*P*<0.05). Exogenous IL-8 significantly stimulated cell proliferation in four SCLC cell lines tested in a dose-dependent fashion. Cell proliferation was increased by between 18% (*P*<0.05) and 37% (*P*<0.05). Stimulation of cell proliferation by IL-8 was also demonstrated by analysis of proliferating cell nuclear antigen expression and cell cycle in H69 cells. Furthermore, we investigated which receptor(s) mediated the mitogenic function of IL-8 in lung cancer cells. We found that cell proliferation was significantly reduced by anti-CXCR1 antibody but not by anti-CXCR2 antibody. In conclusion, IL-8 can act as an autocrine and/or paracrine growth factor for lung cancer cells, and the mitogenic function of IL-8 in lung cancer is mediated mainly by CXCR1 receptor.

Lung cancer is the leading cause of cancer-related death in industrialised countries. The majority of patients with lung cancer have incurable advanced disease with very poor therapeutic options, so new treatment approaches such as targeted therapy are urgently needed. The malignant phenotype of lung cancer may be partially attributable to abnormalities in growth factors and their receptors acting via both autocrine and paracrine pathways. An understanding of the abnormal expression of such growth factors and their receptors is crucial to find new therapeutic targets. Lung cancer can be classified into small cell lung cancer (SCLC), which has a neuroendocrine phenotype, and non-small cell lung cancer (NSCLC), which includes adenocarcinoma, squamous cell carcinoma and large cell carcinoma. Both SCLC and NSCLC have been shown to produce a variety of growth factors (Woll, 1996).

Interleukin-8/CXCL8 (IL-8) is a member of the CXC chemokine family, which was originally classified as a neutrophil chemoattractant with inflammatory activity (Baggiolini *et al*, 1989). Two IL-8 receptors, CXCR1 and CXCR2, have been identified. They share 77% amino-acid identity and belong to the superfamily of seven transmembrane domain, G protein-coupled receptors, whose signalling is mediated by heterotrimeric G proteins, resulting in the exchange of GDP for GTP on the *α* subunit of the G protein (Wess, 1997). CXCR1 and CXCR2 have been found on many normal cells such as neutrophils, basophils, lymphocytes, monocytes, keratinocytes and endothelial cells.

Interleukin-8 is a potent angiogenic factor in several cancers including NSCLC (Smith *et al*, 1994) and is associated with metastasis (Singh *et al*, 1994; Ueda *et al*, 1994; Luca *et al*, 1997). Elevated IL-8 is correlated with angiogenesis, tumour progression and poor survival in NSCLC (Yuan *et al*, 2000; Masuya *et al*, 2001; Orditura *et al*, 2002; Chen *et al*, 2003). CXCL8 has been shown to be mitogenic for cancers such as melanoma (Schadendorf *et al*, 1993), colon cancer (Brew *et al*, 2000; Li *et al*, 2001), pancreatic cancer (Miyamoto *et al*, 1998), malignant mesothelioma (Galffy *et al*, 1999) and Kaposi's sarcoma (Masood *et al*, 2001). However, the role of IL-8 in lung cancer has been controversial. It was shown that the inhibition of lung cancer growth by targeting IL-8 in a mouse model was solely due to the inhibition of the angiogenic effect of IL-8 (Arenberg *et al*, 1996). Interleukin-8 was also shown to inhibit directly lung cancer cell proliferation *in vitro* (Wang *et al*, 1996). CXCR1 and CXCR2 were found on a variety of tumour cells, but have not been reported in lung cancer. In this study, we investigated the expression of IL-8 and its receptors, CXCR1 and CXCR2, in a panel of NSCLC and SCLC cell lines and characterised the mitogenic role of IL-8 in lung cancer growth.

## MATERIALS AND METHODS

### Lung cancer cell lines and cell culture

The SCLC cell lines used were CORL-24, GLC-19, H69, H345, H711 and Lu-165. The NSCLC cell lines used were A549, H460 and MOR/P. All cell lines were cultured in RPMI 1640 (BioWhittaker, Verviers, Belgium) and 10% FBS (QB perbio, Tattenhall, Cheshire, UK) in humidified 5% CO_2_, 95% air at 37°C. Conditioned medium was obtained by collecting medium from MOR/P (5 × 10^7^ cells) or H460 (5 × 10^6^ cells) cells cultured in 5 ml of 10% FBS in RPMI 1640 for 48 h.

### Reverse transcription–polymerase chain reaction

The expression of mRNA for IL-8 and its receptors was determined by reverse transcription–polymerase chain reaction (RT–PCR). Total RNA was isolated by using the RNeasy mini kit (Qiagen, West Sussex, UK) following the manufacturer's protocol. A 0.5 *μ*g portion of total RNA was reverse-transcribed for subsequent PCR amplification for each pair of primers in a volume of 10 *μ*l, including 10 U of enhanced AMV reverse transcriptase (Sigma, Poole, Dorset, UK), 20 U of RNase inhibitor (Sigma), 0.5 *μ*g of oligo(dT)_15_ primer, 0.5 nmol of each dNTP and 1 × first-strand buffer (50 mM Tris-HCl, pH 8.3, 40 mM KCl, 8 mM MgCl_2_, 1 mM dithiothreitol) provided by Sigma. The reaction was incubated at 45°C for 50 min. A 10 *μ*l portion of the RT products was then brought to a volume of 50 *μ*l containing 0.2 nmol of each dNTP, 1 U of *Taq* polymerase (Sigma), 0.1 *μ*g of both the upstream and downstream PCR primers and 1 × PCR buffer (10 mM Tris-HCl, pH 8.3, 50 mM KCl, 1.1 mM MgCl_2_, 0.01% gelatin) provided by Sigma. The primers for IL-8 are as follows: sense, 5′-ATG ACT TCC AAG CTG GCC GTG GCT-3′; antisense, 5′-TCT CAG CCC TCT TCA AAA ACT TCT-3′. The primers for CXCR1 are as follows: sense, 5′-CCT TCT TCC TTT TCC GCC AG-3′; antisense, 5′-AAG TGT AGG AGG TAA CAC GAT G -3′; The primers for CXCR2 are as follows: sense, 5′-ATT CTG GGC ATC CTT CAC AG-3′; antisense, 5′-TGC ACT TAG GCA GGA GGT CT-3′. The primers for GAPDH are as follows: sense, 5′-CCA CCC ATG GCA AAT TCC ATG GCA-3′; antisense, 5′-TCT AGA CGG CAG GTC AGG TCC ACC-3′. Amplification was carried out in a Biometra thermal cycler after an initial denaturation at 94°C for 3 min. This was followed by 35 cycles of PCR using the following temperature and time profile: denaturation at 94°C for 40 s, primer annealing at 58°C for 40 s, primer extension at 72°C for 1 min and a final extension of 72°C for 6 min. The PCR products were visualised by electrophoresis on a 2% agarose gel in 0.5 × TBE buffer (44.5 mM Tris borate, 1 mM EDTA, pH 8.3) after staining with 0.5 *μ*g ml^−1^ ethidium bromide.

### Enzyme-linked immunosorbent assay

The expression of IL-8 protein was determined by enzyme-linked immunosorbent assay (ELISA). For collecting samples from culture media, serum-free RPMI medium was used. The media were harvested at the indicated times and stored at −20°C before analysis. The concentrations of IL-8 in the culture media were determined by ELISA (R&D systems, Abingdon, UK) according to the manufacturer's instructions. Briefly, 100 *μ*l samples containing standard amounts of recombinant IL-8 (rIL-8) or study samples were added in triplicate to individual wells and incubated at room temperature for 1 h. After five washes, 100 *μ*l of biotinylated IL-8 antibody diluted in dilution buffer (0.1% bovine serum albumin, 0.05% Tween 20 in Tris-buffered saline (20 mM Trizma-base, 150 mM NaCl)) was added for 1 h. After another five washes, 100 *μ*l of streptavidin–horseradish peroxidase (HRP) conjugate that had been diluted to 1/200 in dilution buffer was added for 30 min. After a final wash, 100 *μ*l of the substrate buffer containing the HRP substrate tetramethylbenzidine dihydrochloride and hydrogen peroxide in 0.05 M phosphate-citrate buffer (pH 5.0) was added for 30 min in the dark and colour developed in proportion to the amount of IL-8 present. The reaction was stopped by adding 100 *μ*l of stop solution (1.8 M sulphuric acid), and the degree of colour that had been generated was determined by measuring the optical density (OD) at 450 nm in a Dynatech MR5000 microplate reader.

### Flow cytometry analysis of cell surface expression of CXCR1 and CXCR2

The cells were washed twice with phosphate-buffered saline (PBS) and then suspended in 100 *μ*l of FACS buffer (2% bovine serum albumin, 2% normal rabbit serum in PBS), and then 2 *μ*g of mouse anti-human monoclonal anti-CXCR1 or anti-CXCR2 antibody or IgG control antibody (R&D systems) was added and incubated for 40 min on ice. After washing twice with PBS, the cells were suspended in 100 *μ*l of FACS buffer plus 1 : 20 diluted FITC-conjugated rabbit anti-mouse IgG (DAKO, Denmark) and incubated for 30 min on ice. The cells were washed twice with PBS and fixed in 100 *μ*l of FACS buffer containing 1% paraformaldehyde. The cells were analysed on a FACSort flow cytometer (Becton Dickinson, San Jose, CA, USA).

### Staining of proliferating cell nuclear antigen and DNA

The cells were suspended in 100 *μ*l of PBS and then 500 *μ*l of lysing buffer (0.5% Triton X-100, 0.2 *μ*g ml^−1^ EDTA, 1% BSA in PBS) was added for 15 min on ice. The cells were fixed with 3 ml of 100% ice-cold methanol for 10 min, washed once with PBS, and then 2 *μ*g of mouse anti-human monoclonal anti-proliferating cell nuclear antigen (PCNA, clone PC 10, Sigma) or control mouse IgG_2a_ antibody (R&D systems) diluted in lysing buffer was added and incubated for 30 min at room temperature. After washing twice with PBS, the cells were suspended in 100 *μ*l of lysing buffer plus 1 : 20 diluted FITC-conjugated rabbit anti-mouse IgG (DAKO, Denmark) and incubated for 30 min. The cells were washed once with PBS and 200 *μ*l of DNA-staining solution (10 *μ*g ml^−1^ propidium iodide, 0.2 mg ml^−1^ RNase, 0.1% Triton X-100 in PBS) was added at room temperature for at least 15 min. The cells were analysed on a FACSort flow cytometer.

### MTT assay

Cell proliferation was measured by 3-(4,5-dimethylthiazol-2-yl)-2,5-diphenyltetrazolium bromide (MTT) assay (Sigma, Dorset, England). A total of 5 × 10^3^ cells were seeded into 96-well flat-bottomed plates in triplicate in 100 *μ*l RPMI containing 10% FCS. After overnight culture, cells were treated with various concentrations of rIL-8, monoclonal anti-IL-8, monoclonal anti-CXCR1, monoclonal anti-CXCR2 or control antibody – mouse IgG (R&D systems) – and conditioned medium of MOR/P and H460 for 48 h. A 10 *μ*l portion of MTT (5 mg ml^−1^) was added to each well 4 h before the end of experiments. Then, MTT solvent (100 *μ*l of 0.1 N HCl in anhydrous isopropanol) was added and absorbance of the converted dye was measured at a wavelength of 570 nm. The background was also measured at 690 nm.

### Statistical analysis

All results are expressed as mean±s.d. The unpaired Student's *t*-test was used to evaluate the significance of differences between groups, accepting *P*<0.05 as the level of significance.

## RESULTS

### Constitutive production of IL-8 in lung cancer cell lines

**Figure 1:**
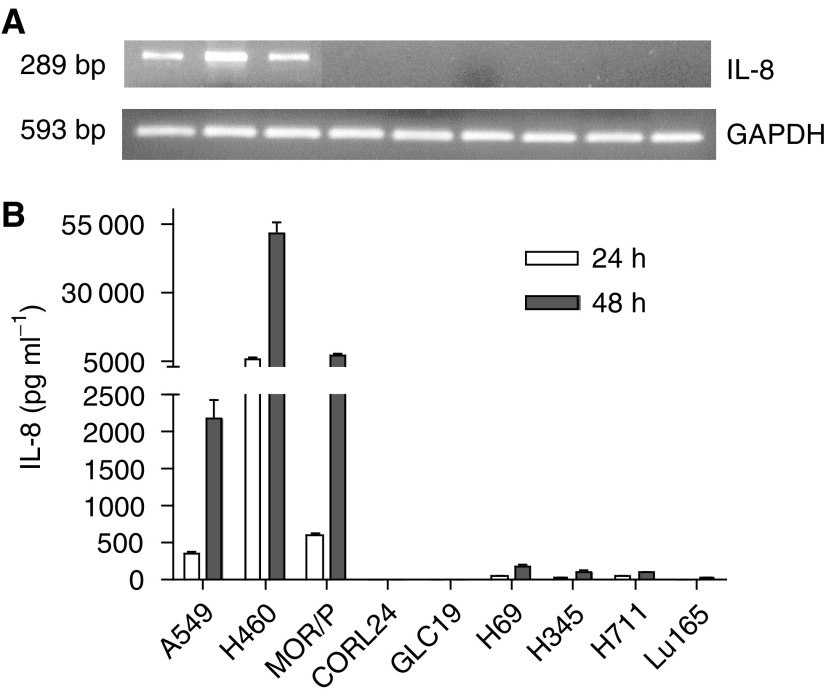
Expression of IL-8 mRNA and protein in lung cancer cell lines. Expression of IL-8 mRNA was measured by RT–PCR (**A**). A 1 *μ*g portion of total RNA was reverse-transcribed for PCR reactions of IL-8 and control GAPDH. The expected 289 bp band of IL-8 mRNA was strongly expressed in A549, H460 and MOR/P, but was undetectable in all SCLC cell lines. Production of IL-8 protein was measured by ELISA (**B**). Conditioned medium was collected after 1 × 10^6^ cells were cultured in serum-free RPMI medium for 48 h. Each bar is the mean±s.e. of three determinations from two independent experiments.

Expression of IL-8 mRNA was detected by RT–PCR, and was found expressed in all NSCLC cell lines: A549, H460 and MOR/P. Although IL-8 mRNA was weakly expressed in the SCLC cell lines H69, H345 and H711, it was not expressed in Lu165, CORL24 and GLC-19 cell lines (Figure 1A

). To assess protein production, IL-8 was measured by ELISA in the supernatants of unstimulated cells cultured in serum-free medium. No detectable IL-8 was found in all cell lines after 6 h of culture. In all three NSCLC cell lines, IL-8 was detected after 24 h, H460 cells produced 6153 pg ml^−1^ 10^6^ cells^−1^, MOR/P 584 pg ml^−1^ 10^6^ cells^−1^ and A549 357 pg ml^−1^ 10^6^ cells^−1^. After 48 h, IL-8 levels were increased to 51 282 pg ml ^−1^10^6^ cells^−1^ in H460, 7299 pg ml^−1^ 10^6^ cells^−1^ in MOR/P and 2053 pg ml^−1^ 10^6^ cells^−1^ in A549. However, IL-8 secretion by SCLC cells was almost negative at 24 h and detectable in only three of six SCLC cell lines at 48 h: 162 pg ml^−1^ 10^6^ cells^−1^ in H69, 109 pg ml^−1^ 10^6^ cells^−1^ in H345 and 100 pg ml^−1^ 10^6^ cells^−1^ in H711 (Figure 1B).

### Expression of CXCR1 and CXCR2 in lung cancer cells

**Figure 2:**
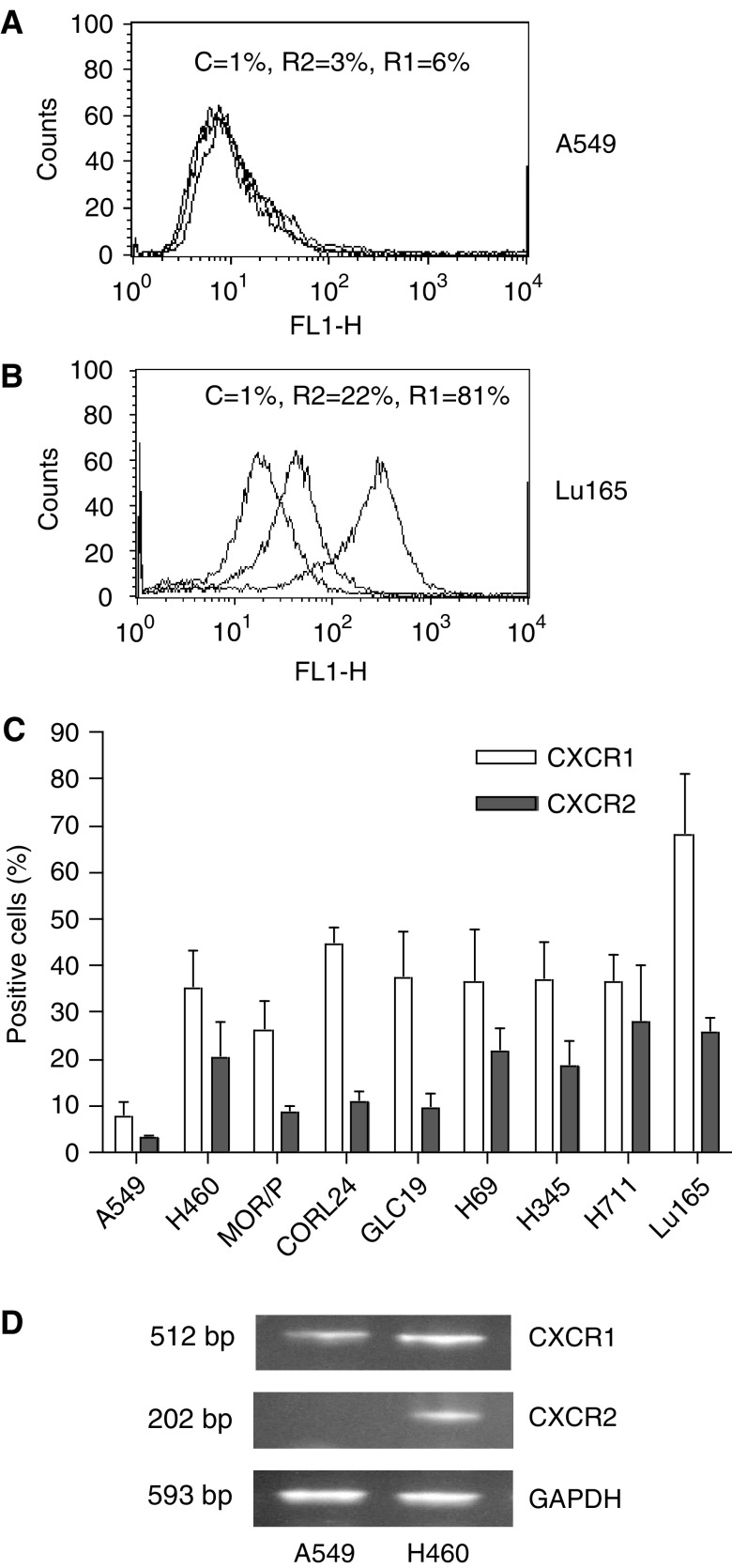
Expression of CXCR1 and CXCR2 in lung cancer cell lines. Expression of CXCR1 and CXCR2 proteins on the cell surface was measured by flow cytometry with mouse anti-human CXCR1 (R1) and CXCR2 (R2) and mouse IgG as control (C). Representative flow cytometric histograms of A549 and Lu165 showing the low expressions of CXCR1 and CXCR2 in A549 (**A**) and high expressions of CXCR1 and CXCR2 in Lu165 (**B**), respectively. Percentage of positive cells of CXCR1 and CXCR2 in nine lung cancer cell lines are summarised in (**C**). Each bar is the mean±s.e. of four independent experiments. Expression of CXCR1 and CXCR2 mRNA was measured by RT–PCR in A549 and H460 (as a positive control) (**D**). Total RNA (1.5 *μ*g) was reverse-transcribed for PCR reactions of CXCR1, CXCR2 and control GAPDH. The expected 512 bp band of CXCR1 was expressed in A549 and H460. The expected 202 bp band of CXCR2 was expressed in control cell H460 but not in A549 cells.

Expression of IL-8 receptors, CXCR1 and CXCR2, was assessed by flow cytometry and RT–PCR (Figure 2

). Expression of CXCR1 and CXCR2 protein on the cell membrane was measured by flow cytometry (Figure 2A and B). In the SCLC cell lines, the number of CXCR1-positive cells in Lu165 was 68%, followed by CORL24 with 45% and then the other four cell lines with between 36 and 38%. In NSCLC cell lines, the number of CXCR1-positive cells was 35% in H460 and 26% in MOR/P, and a few positive cells were found in A549. In contrast to CXCR1, expression of CXCR2 was much lower in all cell lines tested, ranging from 28% in H711 to 9% in MOR/P. The NSCLC cell line A549 was negative for CXCR2 protein (Figure 2C). We further compared the mRNA levels of CXCR1 and CXCR2 in A549 and H460. CXCR1 mRNA was present in A549 but at a lower level than in H460. CXCR2 mRNA was not present in A549 but in H460 as expected (Figure 2D).

### Interleukin-8 is an autocrine growth factor in H460 and MOR/P cells

**Figure 3:**
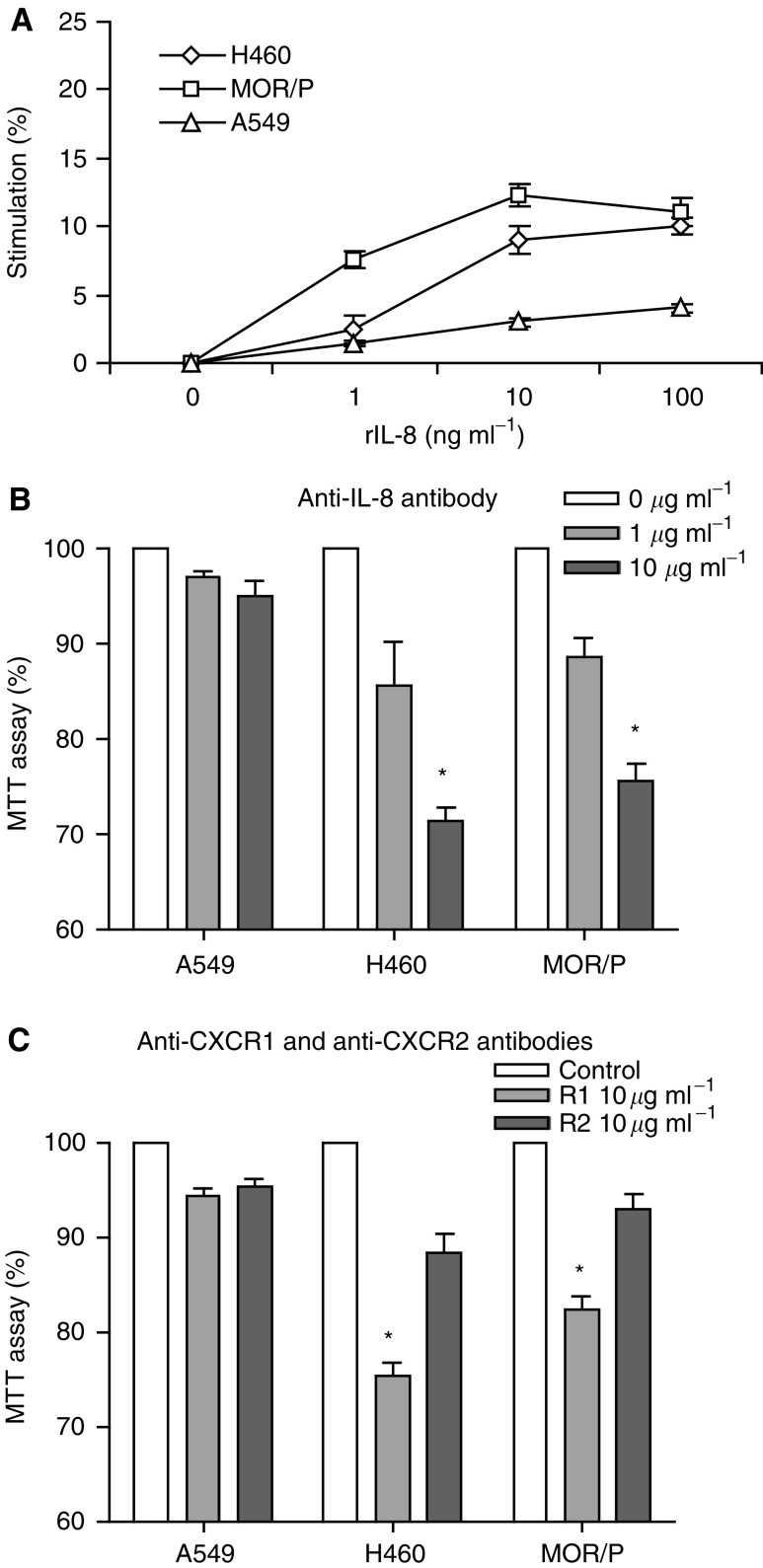
Interleukin-8 is an autocrine growth factor in H460 and MOR/P cells. (**A**) Non-small cell lung cancer cells were treated with rIL-8 (at a concentration of 0, 1, 10 and 100 ng ml^−1^) for 48 h. Cell proliferation was measured by MTT assay. Each point is the mean±s.e. of three determinations from two independent experiments. (**B**) Non-small cell lung cancer cells were treated with anti-IL-8 neutralising antibody (1 or 10 *μ*g ml^−1^) for 48 h. Cell proliferation was measured by MTT assay. Each bar is the mean±s.e. of three determinations from two independent experiments. ^*^P<0.05. (**C**) Non-small cell lung cancer cells were treated with anti-CXCR1 or anti-CXCR2 neutralising antibody (10 *μ*g ml^−1^) or control antibody (mouse IgG at 10 *μ*g ml^−1^) for 48 h. Cell proliferation was measured by MTT assay. Each bar is the mean±s.e. of three determinations from two independent experiments. ^*^P<0.05.

Although all the NSCLC cell lines produced IL-8, only two of them (H460 and MOR/P) expressed IL-8 receptors, as there were almost no detectable CXCR1 and CXCR2 proteins on the surface of A549. Consistent with this, cell proliferation was enhanced by exogenous rIL-8 in H460 and MOR/P, but not in A549 (Figure 3A

). We also tested whether expression of IL-8 receptors was associated with cell proliferation. When NSCLC cells were 50% confluent, 36% cells were in G_1_ phase and 64% cells were in S and G_2_/M phases. In contrast, 67% cells were in G_1_ phase and 33% cells were in S and G_2_/M phases when cells were 100% confluent. The number of positive cells with CXCR1 or CXCR2 was significantly higher in 50% confluent cells than in 100% confluent cells. Positive cells with CXCR1 and CXCR2 were increased to 43 and 28% from 22 and 6% (both *P*<0.01), respectively, in H460. Positive cells with CXCR1 and CXCR2 were increased to 33 and 11% from 21 and 6% (both *P*<0.01), respectively, in MOR/P (Table 1

**Table 1:** Correlation of expression of CXCR1 and CXCR2 with cell proliferation

	**H460**		**MOR/P**	
	**100% confluency**	**50% confluency**	**100% confluency**	**50% confluency**
CXCR1 (%)	22.6±1.7	43.5±3.3*	21.0±1.6	33.0±1.7*
CXCR2 (%)	6.1±0.5	28.2±2.4*	6.2±0.4	11.0±0.6*

* *P*<0.01.

).

To test whether IL-8 acted as an autocrine growth factor in these two cell lines, cells were cultured in the presence or absence of anti-IL-8 antibody. Cell growth was inhibited in a dose-dependent fashion by adding anti-IL-8 antibody. At a concentration of 10 *μ*g ml^−1^ of anti-IL8 antibody, cell proliferation was significantly reduced to 71% (*P*<0.05) and 76% (*P*<0.05) in H460 and MOR/P, respectively. In contrast, cell proliferation was not significantly changed in A549 (Figure 3B). To determine which IL-8 receptor mediates growth in NSCLC, we examined the effects of anti-CXCR1 and anti-CXCR2 on cell proliferation. Anti-CXCR1 antibody significantly reduced cell proliferation of H460 (to 75%, *P*<0.05) and MOR/P (to 82%, *P*<0.05) at a concentration of 10 *μ*g ml^−1^, whereas anti-CXCR2 antibody did not significantly affect cell proliferation of these cells (Figure 3C). A549, which lack surface receptors, did not respond to either antibody.

### Exogenous rIL-8 stimulates SCLC cell proliferation through mainly CXCR1

**Figure 4:**
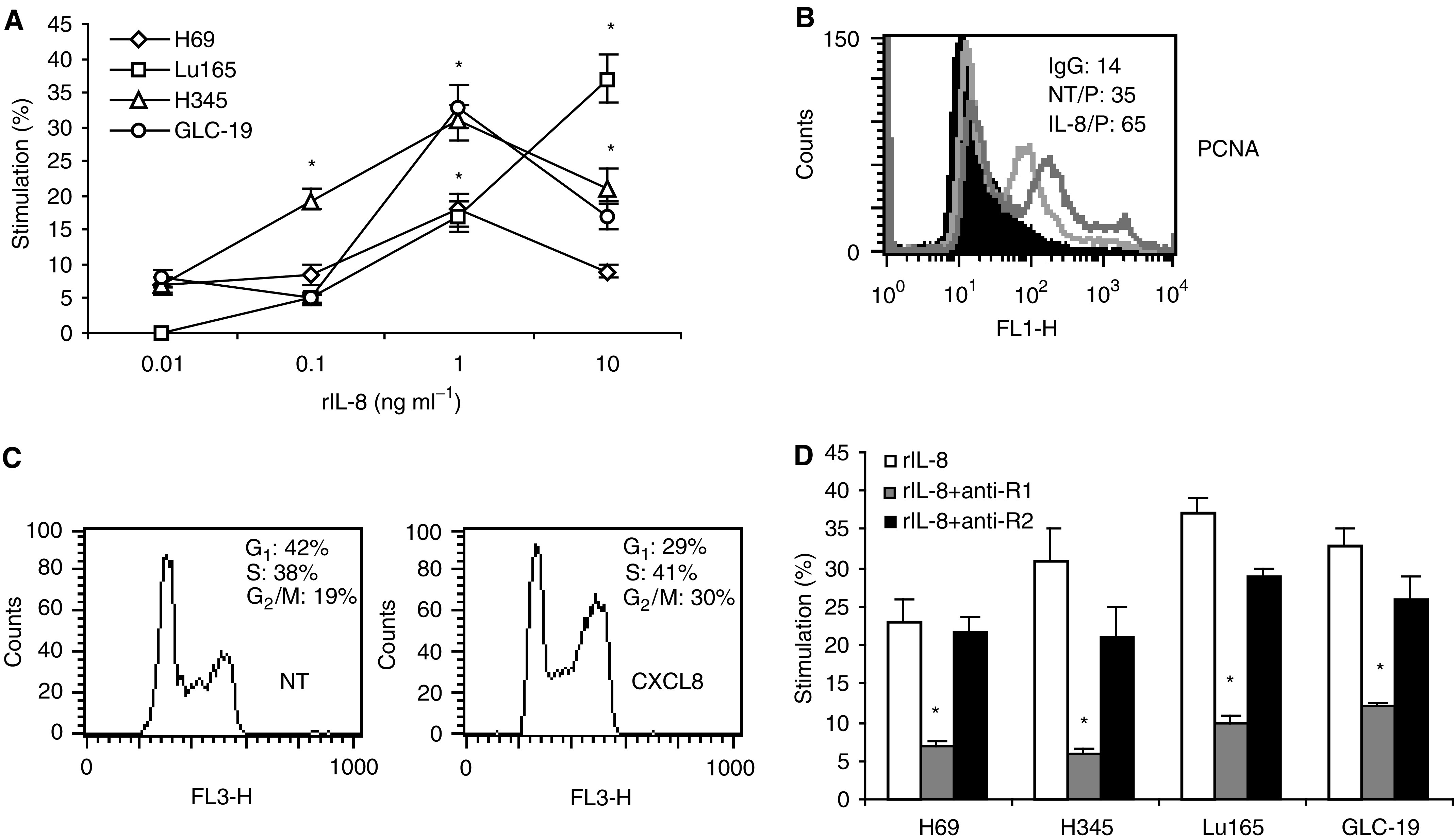
Recombinant IL-8 stimulates SCLC cell proliferation through CXCR1. (**A**) The SCLC cell lines H69, Lu165, H345 and GLC-19 were treated with rIL-8 at concentrations of 0.01, 0.1, 1 and 10 ng ml^−1^ for 48 h. Cell proliferation was measured by MTT assay. Each point is the mean±s.e. of three determinations from two independent experiments. ^*^P<0.05. (**B**) Detection of PCNA intensity by flow cytometry after H69 cells was treated with rIL-8 (1 ng ml^−1^) for 48 h. (**C**) Analysis of DNA content by flow cytometry after H69 cells was treated with rIL-8 (1 ng ml^−1^). (**D**) Small cell lung cancer cells were treated with rIL-8 plus anti-CXCR1 or anti-CXCR2 antibody for 48 h. The concentration of rIL-8 for each cell line was chosen for maximal stimulation: 10 ng ml^−1^ for Lu165 and 1 ng ml^−1^ for others. The final concentration of anti-CXCR1 or anti-CXCR2 was 10 *μ*g ml^−1^. Each bar is the mean±s.e. of three independent determinations of two experiments. ^*^P<0.05 for inhibition of rIL-8-induced proliferation by anti-CXCR1 antibody.

Because SCLC cell lines expressed IL-8 receptors but did not secrete IL-8, it could not act as an autocrine growth factor for these cells. However, exogenous rIL-8 stimulated SCLC cell proliferation in a dose-dependent fashion (Figure 4

). Maximal stimulation was obtained in three out of four tested cell lines at a concentration of 1 ng ml^−1^. Cell growth was increased by 33% (*P*<0.05) in GLC-19, 31% (*P*<0.05) in H345 and 18% (*P*<0.05) in H69. Cell proliferation of Lu165 was increased to 37% (*P*<0.05) with rIL-8 at 10 ng ml^−1^ (Figure 4A). To support this result, expression of PCNA, a marker for proliferating cells, and cell cycle were analysed in H69 cells. After IL-8 stimulation (1 ng ml^−1^), the mean fluorescent intensity (MFI) of PCNA was increased to 65 from 35 in H69 (Figure 4B); the G_1_ phase was decreased to 29% from 43%, and S and G_2_/M phases were increased to 41 and 30% from 38 and 19%, respectively (Figure 4C). To determine which IL-8 receptor was involved in IL-8 stimulation, cells were treated with either anti-CXCR1 (10 *μ*g ml^−1^) or anti-CXCR2 (10 *μ*g ml^−1^) and rIL-8 for 48 h. Anti-CXCR1 antibody significantly reduced the proliferation stimulated by IL-8 in all SCLC cell lines (*P*<0.05). However, anti-CXCR2 antibody did not significantly reduce the proliferation (Figure 4D), indicating that the mitogenic effects of IL-8 in SCLC are mainly mediated by the CXCR1 receptor.

## DISCUSSION

Interleukin-8 has been shown to be an important mitogenic factor in a variety of cancers including melanoma (Schadendorf *et al*, 1993; Singh *et al*, 1994), colon cancer (Brew *et al*, 2000; Li *et al*, 2001), pancreatic cancer (Miyamoto *et al*, 1998), malignant mesothelioma (Galffy *et al*, 1999) and Kaposi's sarcoma (Masood *et al*, 2001). The role of IL-8 in lung cancer has not been fully defined. Although there are several reports on lung cancer-derived IL-8 and its effect on tumour growth, their results remain controversial and unclear. Wang *et al* (1996) reported that exogenous rIL-8 and cancer-derived IL-8 inhibited lung tumour proliferation by both autocrine and paracrine pathways in A549 and four other lung cancer cell lines. However, Arenberg *et al* reported that tumour-derived IL-8 directly correlated with the rate of growth of the two human NSCLC cells lines A549 and Calu-1 in SCID mice. Interleukin-8 promoted human lung cancer growth through its angiogenic properties. Interleukin-8 was not found to behave as an autocrine growth factor for the proliferation of NSCLC cells (Arenberg *et al*, 1996). The expression of CXCR1 and CXCR2 was not investigated in these reports. The present study has investigated the expression of both IL-8 and its receptors simultaneously for the first time in a panel of lung cancer cell lines. We found that NSCLC cells produced significant amounts of IL-8. However, SCLC cells produced low or undetectable levels of IL-8. These results were in agreement with previous studies. Many NSCLC cells including A549 and H460 were shown to produce IL-8 (Arenberg *et al*, 1996; Yatsunami *et al*, 1997; Anderson *et al*, 2000; Chen *et al*, 2003) and SCLC cells including H345 were shown to produce low or undetectable levels of IL-8 (Yatsunami *et al*, 1997).

Since the expression of CXCR1 and CXCR2 has not been studied in lung cancer cells, we next examined the expression of these receptors on cell surface by flow cytometry. In NSCLC cells, CXCR1 and CXCR2 were found on H460 and MOR/P, but very few CXCR1 and no CXCR2 were found on A549. By RT–PCR, A549 expressed CXCR1 mRNA but not CXCR2 mRNA. In SCLC cells, CXCR1 and CXCR2 were expressed in all cell lines. SCLC cells responded to exogenous rIL-8 in a dose-dependent fashion. Cell proliferation reached a peak at an rIL-8 concentration of 1 ng ml^−1^ in most cell lines, but cell proliferation reached a peak at an rIL-8 concentration of 10 ng ml^−1^ in Lu165. This may reflect the fact that Lu165 cell expressed more receptors, especially CXCR1, than other cells. These results indicated that IL-8 could act as a paracrine growth factor in SCLC cells.

The finding that IL-8 and its receptors are coexpressed in H460 and MOR/P cell lines led us to speculate that IL-8 could act as an autocrine growth factor in these cells. We demonstrated that cell growth was stimulated by exogenous rIL-8 in H460 and MOR/P, but not in A549. Also, cell growth was significantly inhibited in a dose-dependent fashion by adding anti-IL-8 neutralising antibody to 71 and 76% in H460 and MOR/P, respectively. In contrast, cell proliferation was only slightly decreased in A549. These results suggest that IL-8 is an autocrine growth factor in H460 and MOR/P, but not in A549 NSCLC cells because they lack receptors. This may explain why IL-8 was not found to act as an autocrine growth factor in A549 cells in a previous study (Arenberg *et al*, 1996).

We further investigated which IL-8 receptor(s) mediated the mitogenic function of IL-8 in lung cancer cells. The biological activity of IL-8 is mediated by binding to two closely related receptors, CXCR1 and CXCR2. CXCR1 binds only IL-8 and CXCL6 (granulocyte chemotactic protein-2, GCP-2), but CXCR2 binds all known angiogenic CXC chemokines that contain the Glu-Leu-Arg (ELR) motif including IL-8 (Lee *et al*, 1992). Our results showed that all the lung cancer cell lines tested expressed CXCR1 and CXCR2 except A549. In these cell lines, expression of CXCR2 protein was much weaker than CXCR1. The role of each of these receptors in IL-8-mediated activity remains controversial. Previous studies have shown that the angiogenic effects of IL-8 in human microvascular endothelial cells and NSCLC were mediated by CXCR2 (Addison *et al*, 2000; Salcedo *et al*, 2000; Heidemann *et al*, 2003). The chemotactic response of melanoma cells to IL-8 was mediated by CXCR1 (Ramjeesingh *et al*, 2003), and the mitogenic activity of IL-8 was mediated by both CXCR1 and CXCR2 in colon cancer (Li *et al*, 2001), but only by CXCR1 in monocytes (Browning *et al*, 2000). Our results show that CXCR1 is the major receptor that mediates the mitogenic function of IL-8 in lung cancer. Also, there was evidence of crosstalk between the IL-8 and epidermal growth factor (EGF) receptors. Venkatakrishnan *et al* (2000) reported that in ovarian cancer cells the signal through CXCR1 and CXCR2 by IL-8 transactivated EGF receptor, which is an effective therapeutic target of many EGF receptor inhibitors in NSCLC. Overall, we have shown that IL-8 can function as an autocrine and/or paracrine growth factor in lung cancer cells. Therefore, targeting both IL-8 production and CXCR1 expression may contribute to control of lung cancer progression, invasion and metastasis through both angiogenic and mitogenic properties of IL-8.
